# Molecular Cloning and Functional Analysis of Lumbricin in *Urechis unicinctus*

**DOI:** 10.3390/biology15181590

**Published:** 2026-09-09

**Authors:** Xiuling Sun, Lihuan Sun, Mengjing Fan, Yuxin Li, Litao Zhang

**Affiliations:** School of Life Sciences, Jining Medical University, Rizhao 276800, China; hatchslack@163.com (X.S.); 15506687752@163.com (L.S.); 13583460528@163.com (M.F.); liyuxin_0424@163.com (Y.L.)

**Keywords:** *Urechis unicinctus*, lumbricin, LPS (lipopolysaccharide) challenge, antibacterial activity

## Abstract

Lumbricin, a classic proline-rich antimicrobial peptide (PrAMP), has been primarily characterized in terrestrial clitellates, with limited reports in other annelid lineages, leaving a critical knowledge gap regarding its presence and immune roles in marine invertebrates. The echiuran worm *Urechis unicinctus* lives in microbe-laden intertidal mudflats and depends on innate immunity against pathogens, yet its lumbricin homologs have remained unreported. In this study, we aimed to clone full-length lumbricin cDNA and systematically characterize its molecular traits, tissue distribution, LPS (lipopolysaccharide)-triggered transcription and in vitro antibacterial function. Sequence analysis showed the deduced 67-amino-acid *U. unicinctus* lumbricin contained 10.4% proline and lacked a signal peptide, retaining the conserved N-terminal cationic patch and C-terminal motifs of earthworm lumbricins. Phylogenetic analysis confirmed its close evolutionary linkage to *Eisenia andrei* lumbricin-related peptide. Quantitative PCR (qPCR) detected ubiquitous constitutive expression, peaking in the body wall, followed by the hindgut and midgut. LPS induced rapid 3 h upregulation in the body wall for acute local defense, with massive late-stage elevation at 48 h in the hindgut, anal sacs and coelomic fluid. Recombinant lumbricin exerted broad-spectrum growth-inhibitory effects against Gram-positive and Gram-negative bacteria. This study is the first to identify a functional lumbricin homolog in marine non-clitellate annelids (echiurans), which expands the taxonomic range of the lumbricin family and provides new insights into innate immune defense of marine benthic organisms.

## Simple Summary

Lumbricin is a well-known proline-rich antimicrobial peptide primarily characterized in terrestrial clitellates, with no prior reports in marine invertebrates. Here, we identified a novel lumbricin homolog from the marine benthic worm *Urechis unicinctus*. The deduced 67-amino-acid *U. unicinctus* lumbricin contained 10.4% proline and lacked a signal peptide, retaining the conserved N-terminal cationic patch and C-terminal motifs of the lumbricin family. Its transcripts were most abundant in the body wall and hindgut, which directly contact environmental microbes. Upon bacterial LPS (lipopolysaccharide) stimulation, *lumbricin* gene expression surged dramatically in a tissue- and time-dependent pattern, suggesting a potentially important role in the innate immune defense of *U. unicinctus*. Recombinant lumbricin displayed broad-spectrum antibacterial activity against both Gram-positive and Gram-negative bacteria, with potent growth-inhibitory capacity. Our findings can expand the distribution scope of the lumbricin family and provide evidence supporting its potential role in the innate immune defense of marine non-clitellate annelids.

## 1. Introduction

Antimicrobial peptides (AMPs) are a large family of gene-encoded, ribosomally synthesized bioactive peptides ubiquitous across all living organisms [1]. These endogenous peptides exhibit a wide range of inhibitory effects against invading pathogens, such as enveloped viruses, bacteria, and fungi [2]. Furthermore, they are recognized as key effector molecules of the innate immune system [3]. Moreover, AMPs constitute the primary defensive barrier in all living organisms and counter continuous microbial threats from external environments, maintaining physiological homeostasis and promoting the host’s survival [4]. The latest version of the Antimicrobial Peptide Database (APD, http://aps.unmc.edu/AP/, accessed on 20 July 2026) had cataloged >6309 AMP sequences, acquired from synthetic, natural, and predictive sources, by January 2026. Of these, >3000 natural AMPs were obtained from animal, plant, and microbial taxa [5]. Typical AMPs are structurally short polypeptides comprising 10–100 L-amino acid residues, with net positive charges of +2 to +9 [6]. Moreover, this cationic feature selectively attaches them to pathogen membranes by forming electrostatic interactions with microbial surfaces’ anionic LPS (lipopolysaccharide), thus stimulating membrane pore formation, cytoplasmic leakage, and ultimately microbial lysis [7].

Lumbricin, a distinctive proline-rich antimicrobial peptide (PrAMP), was first purified and functionally characterized from the earthworm *Lumbricus rubellus*. This 62-residue peptide exhibits potent broad-spectrum antimicrobial activity with negligible hemolytic toxicity in vitro [8]. Several studies have identified the homologous sequences of lumbricin in different terrestrial oligochaete species, such as *Metaphire tschiliensis* [9], *Metaphire guillelmi* [10], and *Eisenia andrei* [11]. However, although a small number of studies have characterized lumbricin homologs in non-oligochaete annelids such as medicinal leeches, the vast majority of available lumbricin data focus on terrestrial clitellate annelid species.

Phylogenomic analyses conducted over the past two decades have robustly established that Echiura are deeply nested within Annelida, constituting a clade of marine non-clitellate annelids [12]. *Urechis unicinctus*, a representative echiuran species, inhabits U-shaped burrows in shallow subtidal mudflats and coastal intertidal zones, with natural populations distributed along the coastlines of Korea, China, Russia, and Japan. This species is traditionally used as a classic experimental model for studying toxic stress adaptation and sulfide tolerance in marine benthic communities [13,14,15]. However, studies on endogenous AMPs of *U. unicinctus* are limited. To date, only three bioactive molecules with confirmed antimicrobial activities have been identified: hemoglobin-F-1 [16], tachykinin [17], and lysozyme [18]. Lumbricin orthologs have not been previously identified and functionally characterized in *U. unicinctus*. 

In this study, we conducted full-length cDNA cloning and tissue expression analyses of a *lumbricin* homolog from *U. unicinctus*. The induction patterns of *lumbricin* expression during immune challenge were evaluated. Lumbricin’s in vitro antibacterial activity was also assessed. The results provide a mechanistic insights into lumbricin-mediated antibacterial immunity and furnish a basis for further innate immune research in marine non-clitellate annelids.

## 2. Materials and Methods

### 2.1. Animal Rearing and Immune Stimulation Sampling

Live *U. unicinctus* were acquired from the local aquatic market in Rizhao City, Shandong Province. All specimens were acclimated before the experiments in fully aerated artificial seawater at 18 °C, 26‰ salinity, and pH 7.8 for 72 h without feeding to remove gut residual impurities and alleviate physiological stress.

For basal tissue expression profiling, target tissues and body fluids, such as the body wall, hindgut, anal sacs, midgut, and coelomic fluid, were collected from healthy worms lacking any immune stimulation.

For LPS stimulation analysis, 102 worms (14.4 ± 0.7 cm in length and 60.7 ± 6.0 g in wet weight) were randomly categorized into LPS treatment and PBS blank control groups. The coelomic cavity of each treatment group worm was microinjected with 150 μL of sterile LPS (0.5 mg/mL working solution). This dosage was selected based on published invertebrate immune-challenge data and our pilot assays, to achieve robust immune activation while avoiding undue physiological stress in the experimental animals. The control group worms received an equivalent volume of phosphate-buffered saline (PBS, pH 7.4). Tissues (including the body wall, hindgut, and anal sacs) and coelomic fluid were subsequently sampled at 0, 3, 6, 24, and 48 h post-injection, immediately snap-frozen in liquid nitrogen, and transferred to −80 °C for subsequent quantitative assays. Preliminary trials revealed that worms exhibited progressive physiological deterioration and higher mortality beyond 48 h post-injection, including impaired locomotion and burrowing behavior. We therefore limited formal sampling to 48 h, the last time point producing physiologically stable and reliable data.

### 2.2. Total RNA Isolation and First-Strand cDNA Synthesis

Total tissue RNA was isolated by following the protocol provided in the RNAiso Plus reagent kit (Takara, Shiga, Japan). For reverse transcription, RNA samples with OD260/280 values ranging from 1.8 to 2.0 were selected, and cDNA was generated using the PrimeScript RT reagent Kit containing gDNA Eraser (Takara), which effectively removed residual genomic DNA contamination. The acquired cDNA was aliquoted and placed at −20 °C for gene sequence validation and quantitative real-time PCR (qRT-PCR) assessment.

### 2.3. Mining, Cloning, and Bioinformatic Characterization of Lumbricin from U. unicinctus

Using a local NCBI BLAST+ 2.17.0 homology search, the unannotated homologous sequence of lumbricin was acquired from the previously published *U. unicinctus* transcriptome dataset (NCBI SRA accession: SRA122323). For sequence screening, the *E. andrei* lumbricin amino acid sequence (GenBank: ARR73564.1) was set as the query template. The target fragments were amplified using the custom primers flanking the full-length *lumbricin* open reading frame (ORF), which were cloned into pMD19-T and subjected to bidirectional Sanger sequencing for sequence confirmation.

The ProtParam online server (https://web.expasy.org/protparam/, accessed on 22 July 2026) was utilized to compute multiple physicochemical parameters of the target peptide, including molecular weight, isoelectric point (pI), grand average of hydropathicity (GRAVY), and amino acid composition. SignalP 6.0 (https://services.healthtech.dtu.dk/services/SignalP-6.0/, accessed on 22 July 2026) was adopted to identify potential signal peptide sequences and predict secretory characteristics of the deduced polypeptide. Target full-length ORF sequences were predicted using the NCBI ORF Finder online tool (https://www.ncbi.nlm.nih.gov/orffinder/, accessed on 22 July 2026). Multiple sequence alignments of lumbricin homologs from diverse invertebrate species were performed using Clustal Omega 1.2.4 with default parameters, and validated by ClustalX 2.0 for consistency [19]. Homology searching was performed via BLASTp against the NCBI non-redundant protein database; percent similarity, query coverage and E-value of top homologous hits were recorded to support sequence homology assignment. Residue conservation was calculated using ConSurf (https://consurf.tau.ac.il/, accessed on 10 August 2026), generating conservation scores ranging from 1 (variable) to 9 (highly conserved). A neighbor-joining (NJ) phylogenetic tree was developed utilizing MEGA 12.0 software with 1000 bootstrap replicates to investigate evolutionary associations [20]. To assess topological congruence, a consensus species phylogeny of the six studied taxa was constructed using the phyloT web server (https://phylot.biobyte.de/, accessed on 12 August 2026), which retrieves hierarchical taxonomic relationships from the NCBI Taxonomy database. This species tree was then compared side-by-side with the lumbricin gene tree.

### 2.4. Tissue Distribution and LPS-Induced Expression Pattern Analysis

To quantify the mRNA levels of *lumbricin* in different *U. unicinctus* tissues and at various post-challenge time points, quantitative PCR (qPCR) was carried out on a CFX Connect Real-Time PCR System (Bio-Rad, Hercules, CA, USA). Each qPCR reaction mixture (20 μL) comprised 2× TB Green Premix Ex Taq II (10 μL), reverse and forward primers (each 2 μM; 4 μL), and diluted cDNA template (2 μL). The internal reference for transcript normalization was β-actin (GenBank GU592178.1). The relative mRNA expression was calculated via the 2^−ΔΔCt^ comparative quantification method [21]. Four individuals were selected as biological replicates for each group based on RNA quality and tissue integrity.

### 2.5. Prokaryotic Recombinant Expression, Purification, Antibacterial Functional Assays and Biosafety Assessment

The full *lumbricin* ORF of *U. unicinctus* was amplified using specific primers with EcoR I and Hind III restriction sites. Then, this ORF was digested, inserted into pET-28a, and transformed into *E. coli* BL21 (DE3), and sequence-verified positive transformants were used for subsequent induction. IPTG (isopropyl β-D-1-thiogalactopyranoside) was added to a final 1 mM concentration to induce protein expression. The soluble recombinant protein fused with 6× His tags was acquired with low-temperature incubation at 19 °C for 12 h. The His-tagged target peptide was purified by following the affinity chromatography protocol of the commercial His-Tagged Protein Purification Kit.

The antibacterial capacity of *U. unicinctus* was assessed against four standard bacterial strains, including one Gram-positive strain (*Staphylococcus aureus*) and three Gram-negative species (*Enterobacter aerogenes*, *Proteus vulgaris*, and *Escherichia coli*). All test strains were cultured in liquid LB medium until the OD_600_ absorbance value reached 0.5. Each well of the 96-well microplate was loaded with 50 μL purified recombinant *U. unicinctus* lumbricin (diluted in PBS) and 50 μL bacterial suspension, yielding a total reaction volume of 100 μL and a final peptide concentration of 500 μg/mL. Three control groups were included: blank PBS, small r-His-tag peptide, and size-matched irrelevant r-His-SUMO tag (13 kDa). r-His-SUMO was expressed, purified and assayed at the same concentration as r-Lumbricin to rule out growth interference from molecular crowding or non-specific protein–bacterial membrane interactions. The microplate was incubated at 37 °C with constant orbital shaking at 220 rpm. Each well’s OD_600_ value was automatically assessed every hour for 12 h using a Spark Multimode Microplate Reader (Tecan Group AG, Männedorf, Switzerland) to investigate dynamic bacterial proliferation. 

To quantitatively define the antimicrobial potency of r-lumbricin, two-fold serial dilution broth microdilution MIC assays were performed following standardized CLSI guidelines. A stock solution of purified r-lumbricin was serially diluted in LB medium to generate a concentration gradient ranging from 31.25 μg/mL to 500 μg/mL. Equal volumes (50 μL) of serially diluted peptide and standardized bacterial inoculum were mixed in each well to a final volume of 100 μL. Sterile LB medium served as the sterility control, and bacterial suspension without peptide was set as the growth control. Plates were incubated statically at 37 °C for 16 h. The MIC value was defined as the lowest peptide concentration that completely suppressed visible bacterial growth compared with the untreated growth control. All MIC measurements were conducted in four biological replicates.

For biosafety assessment, hemolytic activity and cytotoxicity assays against human embryonic kidney 293T (HEK-293T) cells were additionally performed. Hemolytic activity was evaluated using freshly isolated sheep erythrocytes. Serial dilutions of r-Lumbricin (0, 10, 25, 50, 100, 250 and 500 μg/mL) were incubated with 2% RBC suspension at 37 °C for 1 h, and absorbance at 540 nm was measured. Triton X-100 (1%) and PBS served as positive and negative controls, respectively. Cytotoxicity against human HEK-293T cells was assessed via CCK-8 assay after 24 h treatment with r-Lumbricin (0, 10, 25, 50, 100, 250 and 500 μg/mL).

### 2.6. Statistical Analysis

All quantitative data are depicted as mean ± SEM. For pairwise significance analysis, multiple group statistical differences were assessed via ANOVA, followed by Duncan’s multiple range test. GraphPad Prism 10 software was employed for all statistical assessments and graph plotting. Differences were deemed statistically significant at a *p*-value < 0.05.

## 3. Results

### 3.1. Molecular Characterization of U. unicinctus Lumbricin

**Figure 1:**
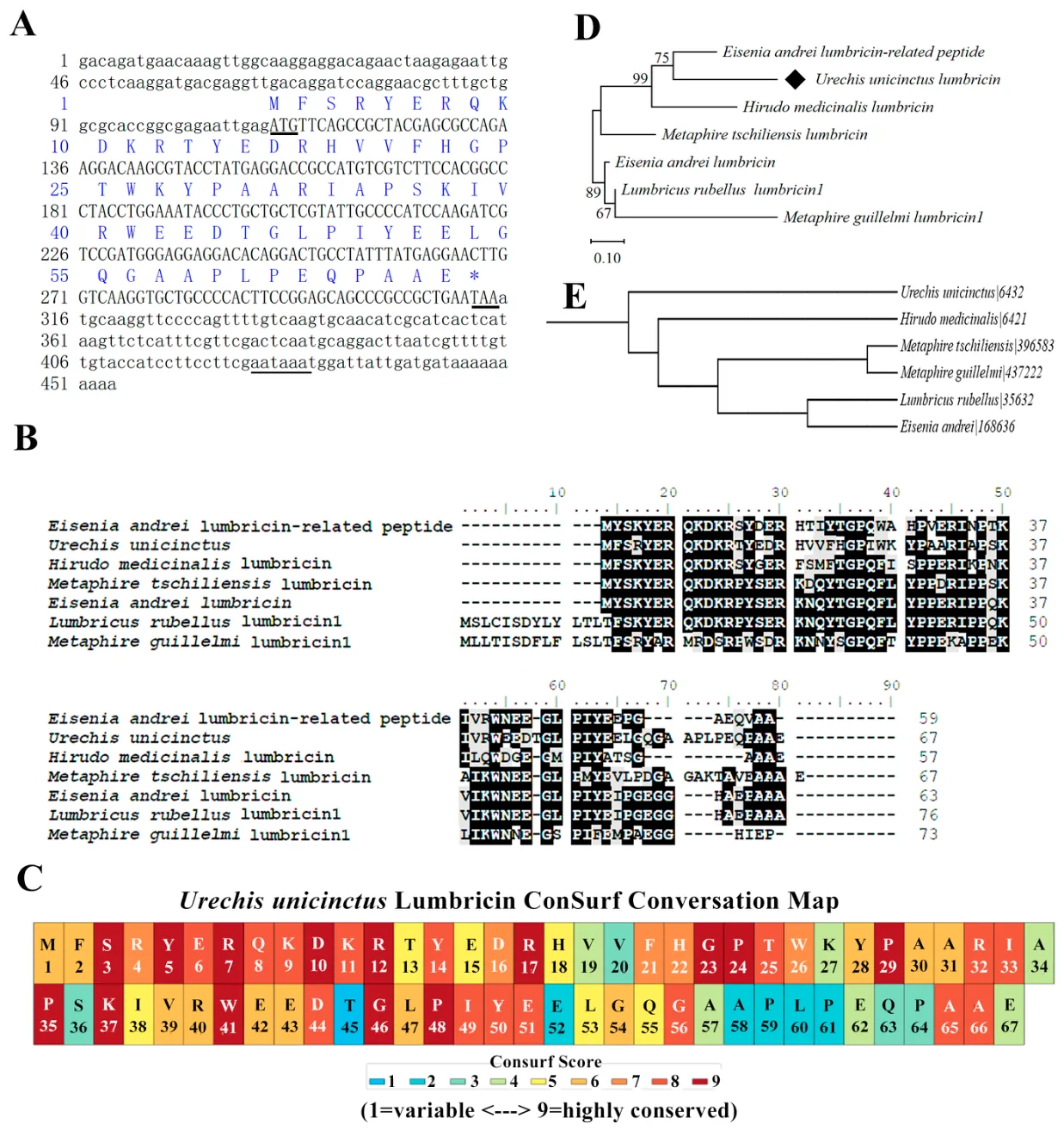
Sequence characterization, residue conservation and phylogenetic comparison of *Urechis unicinctus* lumbricin. (**A**) Nucleotide and deduced amino acid sequences of *U. unicinctus* lumbricin cDNA. The ORF encodes a 67-amino-acid peptide; the start codon (ATG) and stop codon (TAA) are marked. (**B**) Multiple sequence alignment of lumbricin homologs. Identical and conserved residues are shaded black and gray, respectively. (**C**) ConSurf residue conservation map of *U. unicinctus* lumbricin, with scores ranging from 1 (variable) to 9 (highly conserved). (**D**) Neighbor-joining lumbricin gene tree (1000 bootstrap replicates; scale bar = 0.05 substitutions/site; *U. unicinctus* lumbricin marked with a diamond). (**E**) Consensus species phylogeny of the same six taxa generated using phyloT for topological congruence comparison with the lumbricin gene tree. Sequences used in this analysis: *Eisenia andrei* lumbricin (ARR73564.1), *Eisenia andrei* lumbricin-related peptide (ARR73565.1), *Hirudo medicinalis* lumbricin (ABW97520.1), *Lumbricus rubellus* lumbricin1 (AAC64517.1), *Metaphire tschiliensis* lumbricin (AAO16887.1), *Metaphire guillelmi* lumbricin (P86929), and *Urechis unicinctus* lumbricin (this study).

*U. unicinctus lumbricin*’s full-length cDNA was cloned and found to be 454 bp long, comprising a 204 bp ORF encoding a 67-amino-acid polypeptide, a 110 bp 5′ untranslated region (UTR), and a 140 bp 3′ UTR with the canonical polyadenylation signal (AATAAA) at position 423 bp, followed by a poly(A) tail starting at position 444 bp (Figure 1A). ProtParam analysis indicated that the theoretical molecular weight of polypeptide was 7814.77 Da and the isoelectric point (pI) was 6.08. Moreover, the computed grand average of hydropathicity (GRAVY) was −0.954, indicating that the peptide is hydrophilic on average. A residue-level hydropathy plot (Kyte-Doolittle) revealed that while the N-terminal and C-terminal portions are predominantly hydrophilic, the central region contains a short moderately hydrophobic stretch (Supplementary Figure S1).

Multiple sequence alignment demonstrated that *U. unicinctus* lumbricin shared high sequence homology with lumbricin family members from clitellate annelids (Figure 1B). BLASTp analysis confirmed the highest similarity to *E. andrei* lumbricin-related peptide (62.96% similarity, 81% coverage, E-value = 3.18 × 10^−18^). Conserved regions—particularly the N-terminal cationic patch and the C-terminal motif—are present across all aligned sequences, suggesting functional conservation. Within the N-terminal core region (residues 3–51), 14 residues are strictly invariant (S3, Y5, R7, D10, R12, R17, G23, P24, P29, P35, K37, W41, G46, and P48; ConSurf score 9) and 15 are strongly conserved (ConSurf score 8), collectively defining the putative functional domain of the lumbricin family. Notably, the conserved motif SKYERQKDKR (residues 3–12) is enriched in basic residues, consistent with the membrane-targeting mechanism typical of antimicrobial peptides. In contrast, the C-terminal region (residues 52–67) is considerably more variable, likely reflecting species-specific diversification. Interestingly, residue T45 (ConSurf score 1) represents a unique insertion in *U. unicinctus* that is absent in all other aligned homologs, suggesting a lineage-specific structural feature (Figure 1C).

SignalP 6.0 prediction across all lumbricin homologs in our alignment revealed that only *M. guillelmi* lumbricin1 bears a canonical N-terminal signal peptide, and all remaining annelid lumbricin sequences, including the *U. unicinctus* lumbricin characterized here, are signal-peptide-deficient (Table S1).

A phylogenetic tree was developed via the NJ method to investigate the evolutionary association between *U. unicinctus* lumbricin and other known homologs (Figure 1D). The tree revealed that *U. unicinctus* lumbricin and *E. andrei* lumbricin-related peptide were clustered closely, forming a distinct clade separate from other annelid sequences, consistent with the BLASTp analysis. To test topological congruence, we directly compared this lumbricin gene tree with the accepted consensus species phylogeny of the same six taxa (Figure 1E). The gene tree broadly recovered the main annelid clades consistent with organismal evolution, while subtle branching discordance suggested lineage-specific sequence evolution beyond simple neutral tracking of species divergence.

### 3.2. Tissue Expression Analysis of U. unicinctus Lumbricin

**Figure 2:**
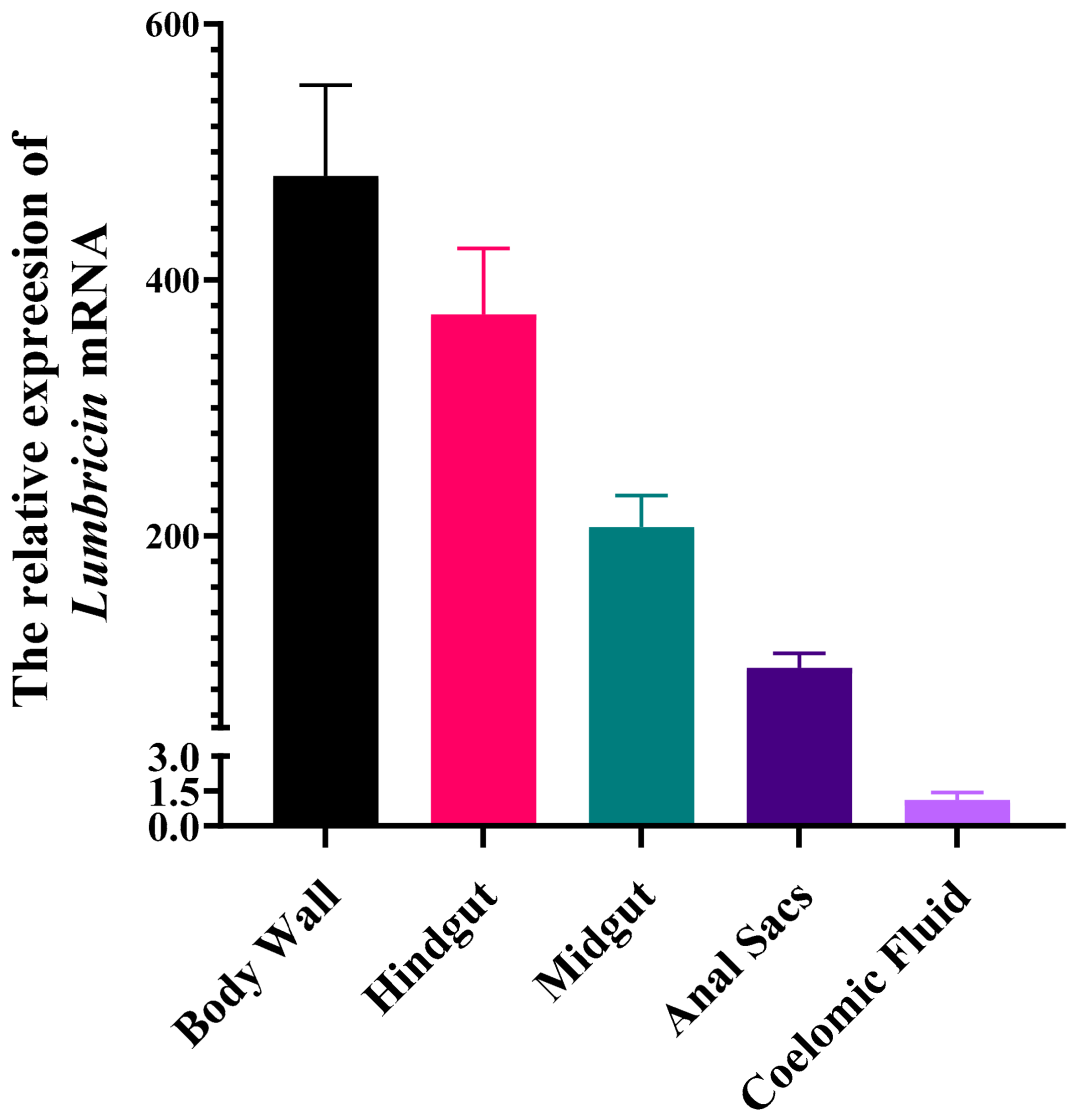
Tissue-biased mRNA expression profiles of *Urechis unicinctus lumbricin*. The relative *lumbricin* expression levels in different tissues (hindgut, body wall, midgut, anal sacs, and coelomic fluid) were evaluated by qPCR. β-actin was set as an endogenous reference gene. Data are depicted as mean ± standard error (n = 4).

The results of tissue expression analysis of *U. unicinctus lumbricin* (Figure 2) indicated that *lumbricin* transcripts were ubiquitously expressed in all tested tissues; however, their expression patterns were distinct. The highest relative expression level was observed in the body wall, followed by the hindgut, midgut, and anal sacs, whereas the coelomic fluid had the lowest transcript abundance.

### 3.3. Expression Profiles of U. unicinctus Lumbricin Post-LPS Stimulation

**Figure 3:**
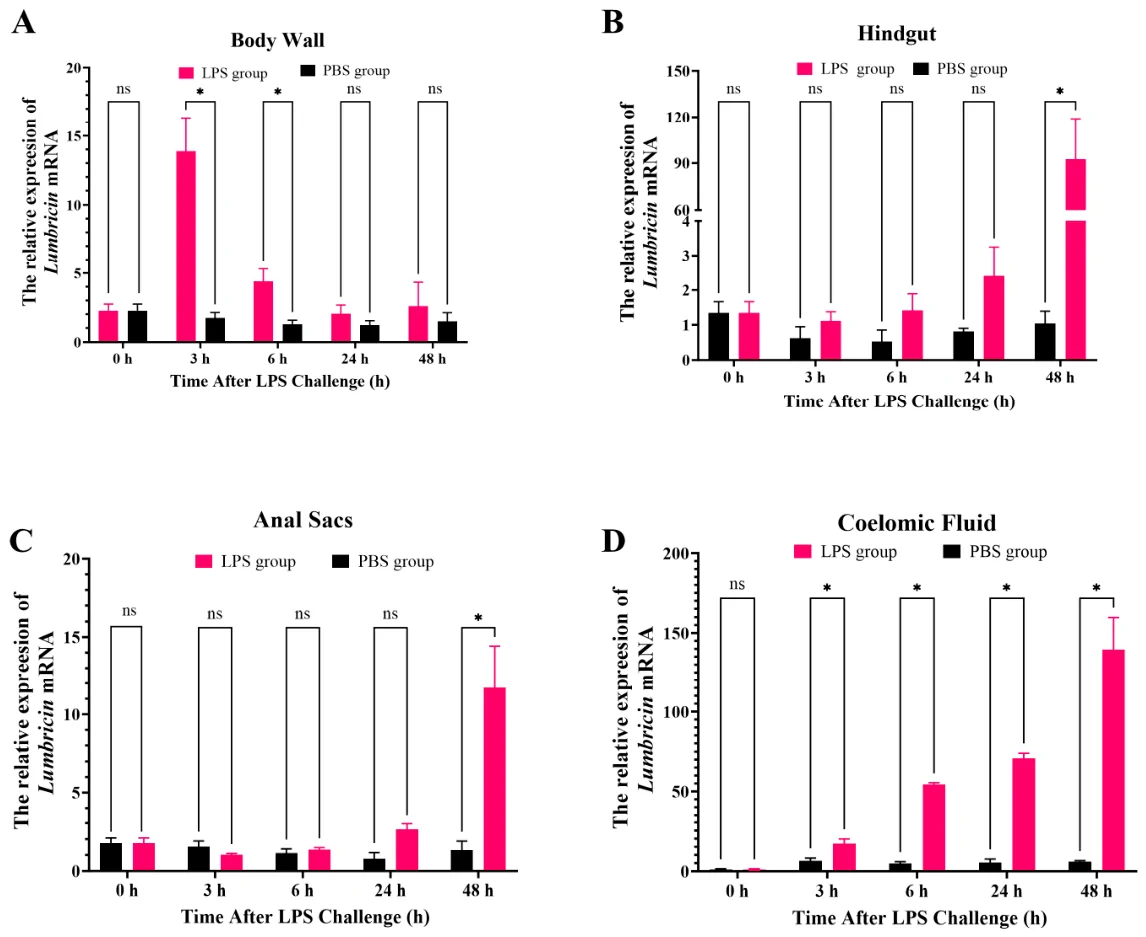
Temporal mRNA expression profiles of *U. unicinctus lumbricin* following LPS and PBS challenge. Relative expression levels of *lumbricin* in the (**A**) body wall, (**B**) hindgut, (**C**) anal sacs, and (**D**) coelomic fluid of *U. unicinctus* were measured at 0, 3, 6, 24, and 48 h post-LPS injection by qPCR. The time point of 48 h represents the final reliable sampling time point. Red bars indicate the LPS-treated group, and black bars denote the time-matched PBS injection control group. The endogenous reference gene was β-actin. Data are denoted as mean ± standard error (n = 4). Statistical comparisons between LPS and corresponding time-matched PBS groups were performed using paired Student’s *t*-test. ns, not significant; * *p* < 0.05.

LPS challenge triggered significant time- and tissue-dependent upregulation of lumbricin transcripts when compared with time-matched PBS injection controls. In the body wall, lumbricin mRNA levels were significantly elevated at 3 h and 6 h post-LPS injection relative to the synchronized PBS group (Figure 3A). In the hindgut and anal sacs, significant induction was only detected at the 48 h timepoint (Figure 3B,C). In coelomic fluid, lumbricin transcript abundance increased progressively over time, with statistically significant upregulation starting at 3 h and peaking at 48 h (24.00-fold relative to the matched PBS control; Figure 3D). It should be noted that 48 h represents the terminal sampling time point limited by animal physiological tolerance, and the continuous upward expression trend observed at this time point does not necessarily indicate the true biological peak of lumbricin transcription.

These findings revealed that LPS transcriptionally activated *U. unicinctus lumbricin* in a tissue- and time-dependent manner, indicating a rapid early induction in the body wall, but a strong, sustained, and late response in the anal sacs, hindgut, and coelomic fluid.

### 3.4. Antibacterial Activity and Biosafety Assessment of Recombinant U. unicinctus Lumbricin

**Figure 4:**
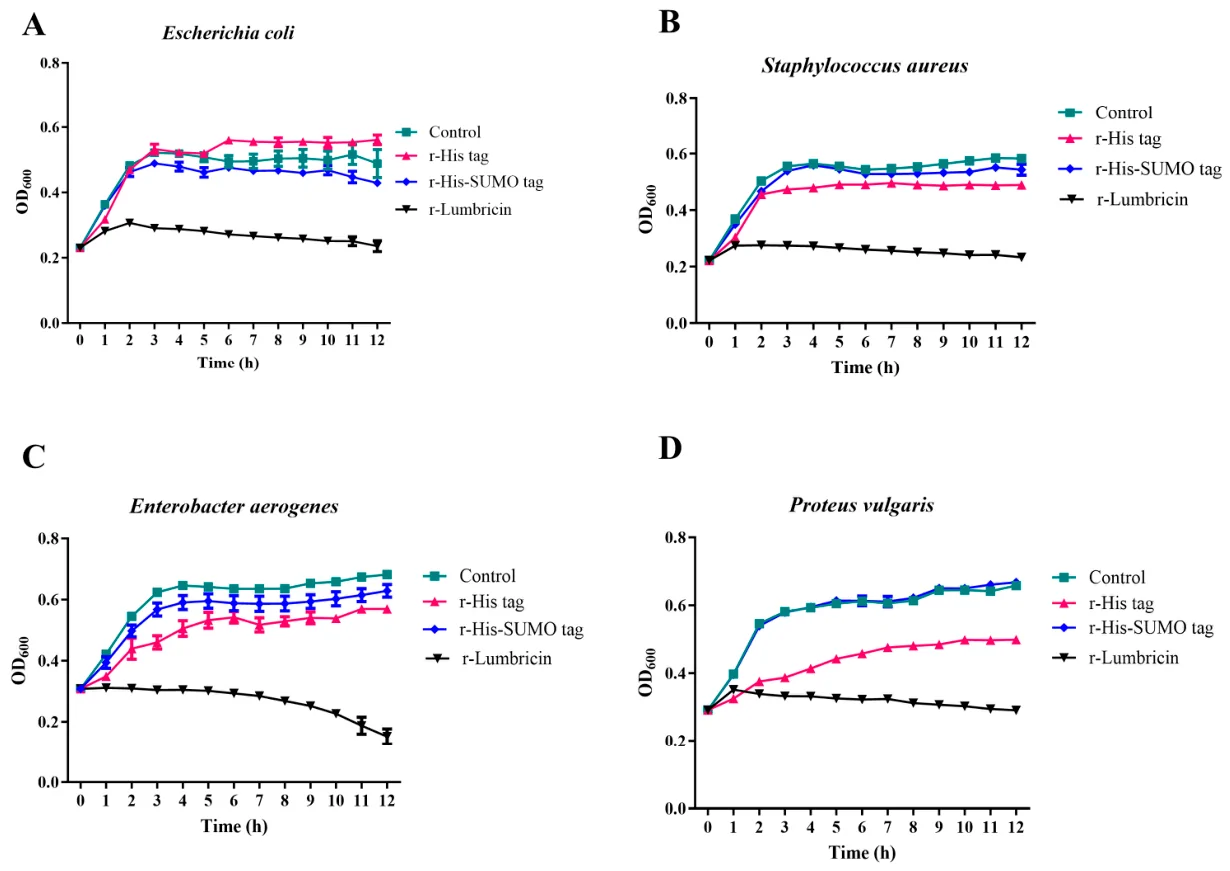
Growth curves of four bacterial strains following treatment with recombinant r-Lumbricin and control proteins. Dynamic proliferation of (**A**) *Escherichia coli*, (**B**) *Staphylococcus aureus*, (**C**) *Enterobacter aerogenes*, and (**D**) *Proteus vulgaris* was monitored by OD_600_ measurement over 12 h. Four groups were included: blank Control, r-His tag, size-matched irrelevant r-His-SUMO tag (13 kDa), and r-Lumbricin. Data are shown as mean ± standard error (n = 4).

SDS-PAGE analysis verified the successful soluble expression of recombinant lumbricin (r-Lumbricin), and the target protein was purified to high homogeneity and subjected to subsequent antibacterial activity evaluation (Supplementary Figure S2). The inhibitory activity of r-Lumbricin was assessed against three Gram-negative bacteria (*Escherichia coli*, *Proteus vulgaris*, and *Enterobacter aerogenes*) and one Gram-positive bacterium (*Staphylococcus aureus*) via a growth curve assay (Figure 4). The blank medium, r-His tag and r-His-SUMO tag groups showed sustained vigorous proliferation of all tested strains within the 12 h incubation period, whereas significant growth inhibition was observed in the r-Lumbricin treatment group across all bacteria. The r-Lumbricin-treated *E. coli* (Figure 4A) specifically showed a small initial rise in OD_600_ before stabilizing at a low value of 0.25, relative to an OD_600_ of around 0.5 in the three control groups. Moreover, a consistent inhibitory pattern was observed in *S. aureus* (Figure 4B), whose OD_600_ remained below 0.28 during r-Lumbricin treatment, whereas the controls reached their peak at 0.58. The strongest bioactivity was found against *E. aerogenes* (Figure 4C), where r-Lumbricin inhibited bacterial multiplication and also promoted a gradual decrease in OD_600_ after early incubation, suggesting anti-bacterial potential, with all the control groups reaching high cell densities corresponding to OD600 values of about 0.68, 0.63 and 0.57, respectively. In *P. vulgaris*, r-Lumbricin significantly restrained its proliferation (Figure 4D), maintaining its OD_600_ at about 0.3 compared to the control’s OD_600_ of 0.66. Further analysis of growth-curve assays revealed strain-dependent differences in growth inhibition. The r-lumbricin produced prominent bacteriostatic effects against *S. aureus*, *E. coli* and *P. vulgaris*. For *E. aerogenes*, treatment with r-lumbricin not only suppressed bacterial multiplication but also resulted in a gradual reduction in culture turbidity over the incubation time. All the results validated that recombinant *U. unicinctus* lumbricin exerts a broad-spectrum in vitro antibacterial effect against both Gram-positive and Gram-negative bacteria.

To quantitatively characterize the intrinsic antimicrobial potency beyond single-concentration growth inhibition, standardized two-fold serial broth microdilution assays were carried out to determine MIC values for each tested strain (Table 1). r-lumbricin exhibited broad-spectrum inhibitory activity against all four bacterial strains. The lowest MIC of 62.5 μg/mL was detected for *E. aerogenes*, consistent with the pronounced reduction in culture turbidity observed in the growth-curve assay. The MIC values for *S. aureus*, *E. coli* and *P. vulgaris* were all 125 μg/mL, confirming robust bacteriostatic activity against these species.

**Figure 5:**
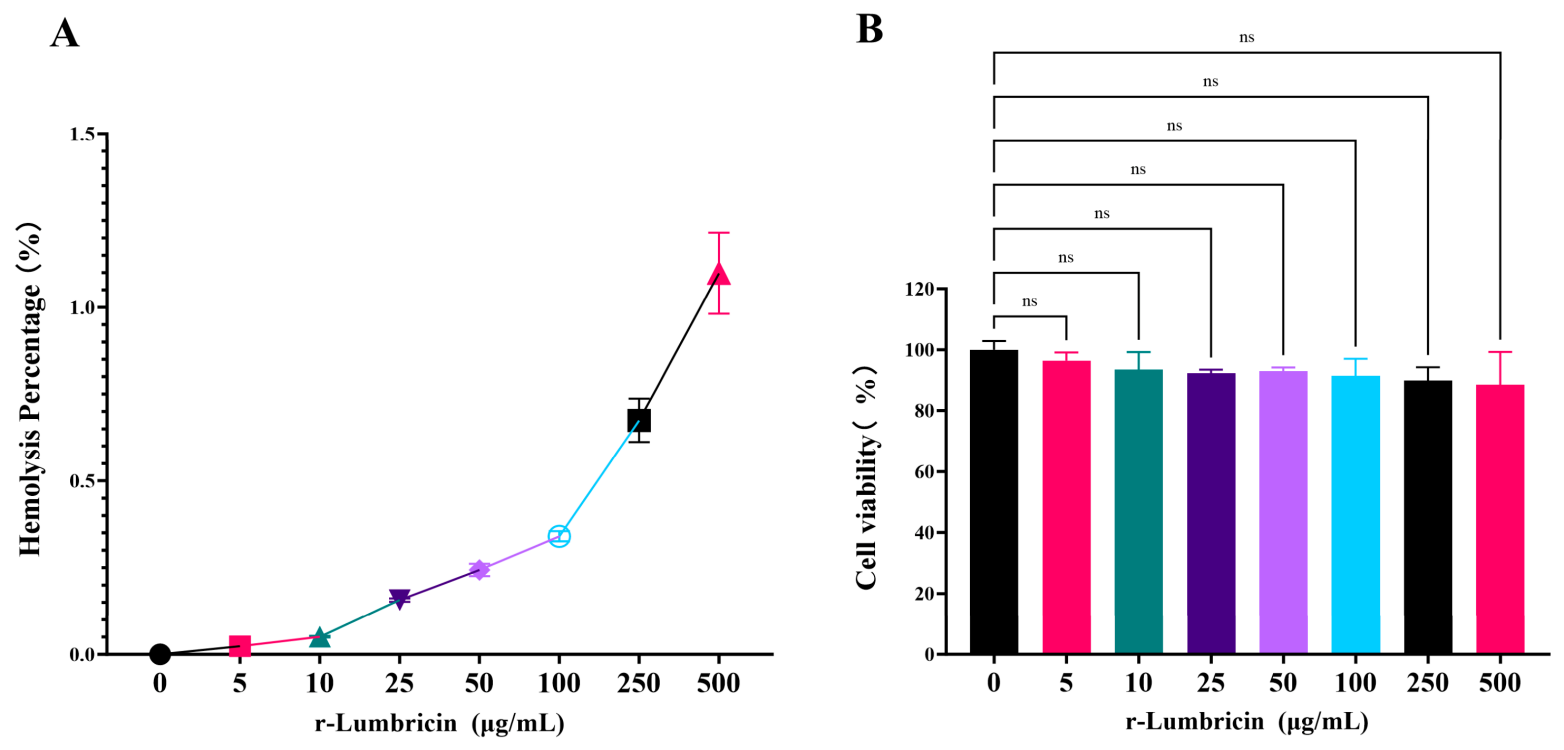
Hemolytic activity and HEK-293T cell viability following treatment with recombinant *Urechis unicinctus* lumbricin. (**A**) Dose–response curve of hemolysis. (**B**) HEK-293T cell viability under gradient peptide concentrations; ns, not significant compared with the untreated control.

The assay to determine the in vitro hemolytic activity of r-Lumbricin showed that at concentrations of 10, 25, 50, 100, 250 and 500 μg/mL, the hemolysis percentages were 0.023, 0.051, 0.157, 0.243 and 0.340%, respectively (Figure 5A). Additionally, incubation with r-Lumbricin at 5–500 μg/mL caused no significant loss of viability in HEK-293T cells, with cell survival consistently exceeding 90% across all tested doses (Figure 5B). Taken together, these data confirm the favorable biosafety profile of *U. unicinctus* lumbricin and support its development as a candidate therapeutic antimicrobial agent.

## 4. Discussion

Antimicrobial peptides are ubiquitous in different organisms and serve as key effectors of innate immunity, particularly in invertebrates [22]. PrAMPs are a unique peptide family that primarily targets intracellular components. Lumbricin is a member of the PrAMP family in annelids [23]. Most characterized lumbricin sequences derive from terrestrial clitellate earthworms such as *L. rubellus* [8], *M. tschiliensis* [9], and *M. guillelmi* [10], with a small number of homologs also recovered from leeches (*E. andrei*) [11]. This study identified and characterized a new lumbricin homolog in the marine echiuran *U. unicinctus*, expanding the documented range of the lumbricin family to marine non-clitellate annelid lineages.

*U. unicinctus* lumbricin comprises 67 amino acids, 10.4% proline content, and 11.9% aromatic amino acid (Phe, Trp, Tyr) content. Although the full length of *U. unicinctus* lumbricin is homologous to that of canonical lumbricins, its proline content is less than the identified average level of 15% for classic lumbricins [23]. This peptide indicated the same proline levels as the lumbricin-related peptide from *E. andrei*, with its theoretical isoelectric point (pI = 6.08) remarkably comparable to that recorded for the latter (6.07) [11]. Although *U. unicinctus* lumbricin possesses a proline proportion of 10.4% (lower than canonical homologs yet within the 10–40% range of PrAMPs), multiple sequence alignment revealed highly conserved N-terminal cationic patch and C-terminal signature motifs shared with other annelid lumbricins (Figure 1B), which firmly supports its classification into the lumbricin family.

Interestingly, SignalP 6.0 analysis confirmed that *U. unicinctus* lumbricin has no canonical N-terminal signal peptide. A systematic screening of all annotated lumbricin homologs further demonstrated that only *Metaphire guillelmi* lumbricin carries a predicted secretion signal; all other family representatives—including sequences from *E. andrei*, *L. rubellus*, *M. tschiliensis*, *H. medicinalis*, and the peptide characterized here—lack this motif, meaning signal peptide absence represents the dominant trait within the lumbricin family. Without an ER–Golgi-targeting signal, this peptide cannot undergo constitutive classical secretion. Instead, it is likely stored intracellularly and exported via leaderless unconventional protein secretion (UPS) upon immune stimulation. This trafficking model aligns with our transcriptional data: lumbricin exhibits minimal basal abundance in coelomic fluid but undergoes robust upregulation at 48 h post-LPS injection, consistent with stimulated release from activated or lysed immune cells. Biotechnologically, the missing signal peptide does not hinder soluble recombinant expression in prokaryotes as shown here, though exogenous secretion tags would be required for eukaryotic secretory production.

Furthermore, phylogenetic analysis confirms that this sequence represents a novel lumbricin homolog. *U. unicinctus* lumbricin clusters within a distinct clade and exhibits close evolutionary relatedness to *E. andrei* lumbricin-related peptide, consistent with our BLASTp results. Side-by-side topological comparison between the lumbricin gene tree and the consensus species phylogeny of the corresponding taxa (Figure 1E) shows that the gene tree largely recapitulates major annelid organismal clades. Nevertheless, subtle branching discordance exists between the two topologies, implying lineage-specific sequence evolution rather than purely neutral tracking of species divergence. This evolutionary pattern reflects the divergence between marine non-clitellate echiurans and terrestrial clitellate annelids, from which most previously characterized lumbricin homologs have been recovered.

In *U. unicinctus*, *lumbricin* mRNA was ubiquitously identified, showing a tissue-biased pattern with highest levels in the body wall, followed by the hindgut and midgut; moderate levels in the anal sacs; and the lowest levels in coelomic fluid (Figure 2). This transcript distribution is consistent with the general AMP pattern observed in invertebrates, where the expression of defense effectors is highest at barrier tissues. Previous studies indicated that in *P. tschiliensis*, lumbricin was restricted to the body wall and lacked intestinal expression [9]; however, in *U. unicinctus*, the digestive tract transcription also suggests immune function, which aligns with the expression profile of lumbricin homologs in *E. andrei* [11]. The high constitutive *lumbricin* transcript abundance measured in the body wall—the tissue that directly interfaces with sediment-associated microbes—supports the hypothesis that this gene might mediate primary barrier immune defense at host–environment interfaces. In *U. unicinctus*, the hindgut is considered a body wall extension, structurally resembles the body wall, and is involved in gas exchange, thus representing another tissue for environmental pathogen exposure [24,25]. Furthermore, it had the second highest *lumbricin* mRNA levels. The midgut is a part of the digestive system that is also in close contact with environmental microorganisms [25], and has the third highest expression. Notably, bulk whole-tissue qPCR cannot discriminate diffuse low transcription from strong expression in specific cell subsets. Follow-up in situ hybridization or immunolocalization assays are needed to resolve lumbricin’s exact cellular distribution for finer functional interpretation.

The LPS stimulation analysis further indicated that *lumbricin* expression is dynamically modulated in a tissue- and time-dependent manner. The early, rapid upregulation of lumbricin in the body wall at 3 h post-LPS stimulation indicated an acute-phase response at the primary barrier to rapidly control invading pathogens at the entrance. The delayed but robust *lumbricin* induction in the hindgut, anal sacs, and particularly the coelomic fluid at 48 h demonstrated a secondary, sustained immune response. The highest expression in the coelomic fluid was 24.00-fold relative to the matched PBS control. Despite its low constitutive expression in this compartment, this striking late upregulation supports the hypothesis that lumbricin could participate in systemic immune responses. Although the induction trend of *lumbricin* resembles that of *thymosin-β12* in *U. unicinctus* [26], its fold changes were much greater, providing transcriptional evidence consistent with distinct immune contributions from this inducible defense effector.

In vitro functional assays verified that r-lumbricin has robust broad-spectrum function against both Gram-positive and Gram-negative bacteria. Complementary standardized MIC assays further quantified its intrinsic antimicrobial potency across a concentration gradient, eliminating limitations of single-dose testing. The strain-specific MIC values revealed stronger activity against *E. aerogenes* (MIC = 62.5 μg/mL) relative to the other three tested strains (MIC = 125 μg/mL), consistent with the bacteriostatic activity observed in growth curve monitoring. These functional traits indicate similar broad-spectrum antimicrobial properties to those of lumbricin isolated from *M. guillelmi* [10]. Conventional lumbricins belong to the PrAMP family, which typically exert antibacterial effects by penetrating the bacterial outer membrane via the self-promoted uptake pathway and binding to intracellular molecular chaperones or ribosomal subunits, thereby inhibiting protein synthesis without causing membrane lysis [23]. By structural analogy, the conserved N-terminal cationic patch of *U. unicinctus* lumbricin likely facilitates initial electrostatic interaction with bacterial LPS, while the C-terminal conserved motif may be involved in intracellular target recognition. The absence of a signal peptide suggests that this peptide may act predominantly in an intracellular compartment or be released via non-classical pathways, potentially serving as a ‘last-resort’ effector against phagocytosed or intracellular pathogens. Compared to other AMP families, PrAMPs, including lumbricins, offer distinct advantages: they are less prone to inducing resistance due to their multiple intracellular targets, exhibit synergism with conventional antibiotics, and display favorable safety profiles. The *U. unicinctus* lumbricin, as a marine-derived PrAMP, may possess unique structural adaptations with higher hydrophilicity that confer stability and activity in marine environments. Its broad-spectrum activity, low cytotoxicity, and lack of hemolytic effects position it as a promising candidate for further therapeutic development, particularly against drug-resistant bacterial infections.

In the present study, we cloned full-length lumbricin cDNA and systematically characterized its molecular features, tissue distribution, LPS-triggered transcription and in vitro antibacterial activity. Nevertheless, this work has several limitations. We only analyzed gene expression at the mRNA level without quantifying lumbricin protein abundance. For our coelomic LPS injection assay, tissue-specific transcriptional upregulation cannot be fully discriminated from uneven LPS tissue accumulation, as we lack fluorescent LPS tracing to quantify tissue LPS loads. Though this confounding factor is partially mitigated by *U. unicinctus*’ open coelomic circulatory system—all tissues are immersed in coelomic fluid, which accounts for the body wall’s rapid 3 h response and delayed 48 h induction in filtration-capable anal sacs—our assumption of rapid LPS mixing driven by worm peristalsis remains unvalidated. We plan to conduct follow-up experiments with fluorescently labeled LPS to disentangle intrinsic tissue immune competence from pharmacokinetic LPS deposition. Furthermore, the exact intracellular antibacterial mechanism of this lumbricin remains unclear; further assays exploring bacterial membrane interactions and target protein identification are required to uncover its detailed molecular mode of action.

## 5. Conclusions

This work is the first to identify a functional lumbricin homolog in marine non-clitellate annelids, expanding the taxonomic range of the lumbricin family. This gene shows tissue-biased constitutive expression at host–environment barrier tissues and exhibits robust, tissue- and time-dependent transcriptional activation upon bacterial LPS exposure. The recombinant peptide exerts broad-spectrum growth-inhibitory effects against both Gram-positive and Gram-negative pathogens. Collectively, these findings improve our understanding of AMPs’ evolution and function in echiurans and provide a basis for further evaluation of the potential applications of this peptide as a new antimicrobial agent.

## Figures and Tables

**Table 1:** Antimicrobial activities of recombinant lumbricin from *Urechis unicinctus*.

Bacteria Name	r-Lumbricin MIC (μg/mL)
**Gram-positive bacteria**	
*Staphylococcus aureus*	125.0
**Gram-negative bacteria**	
*Enterobacter aerogenes*	62.5
*Escherichia coli*	125.0
*Proteus vulgaris*	125.0

## Data Availability

The original contributions presented in this study are included in the article/Supplementary Materials. Further inquiries can be directed to the corresponding author.

## Supplementary Materials

The following supporting information can be downloaded at https://www.mdpi.com/article/10.3390/biology15181590/s1, Figure S1: Kyte–Doolittle hydropathy profile of *Urechis unicinctus* lumbricin predicted using ProtScale; Figure S2: SDS-PAGE detection of recombinant lumbricin expression and purification.; Table S1: SignalP 6.0 prediction of N-terminal signal peptides for all lumbricin homologs.

## Abbreviations

The following abbreviations are used in this manuscript:

AMPs	Antimicrobial peptides
PrAMP	Proline-rich antimicrobial peptide
APD	Antimicrobial Peptide Database
LPS	Lipopolysaccharide
NCBI	National Center for Biotechnology Information
SRA	Sequence Read Archive
ORF	Open reading frame
NJ	Neighbor-joining
qPCR	Quantitative polymerase chain reaction
UTR	Untranslated region
IPTG	Isopropyl β-d-1-thiogalactopyranoside
OD	Optical density
SEM	Standard error of the mean
ANOVA	Analysis of variance
pI	Isoelectric point
GRAVY	Grand average of hydropathicity
PBS	Phosphate-buffered saline
r-Lumbricin	Recombinant lumbricin
