# Supplementary material for: Molecular Cloning and Functional Analysis of Lumbricin in Urechis unicinctus
Source: Biology (Basel). 2026 Sep 9;15(18):1590. doi: 10.3390/biology15181590 (PMC13603904; doi:10.3390/biology15181590)
Supplement: Supplementary file 1 [file biology-15-01590-s001.zip › Figure S1.pdf]

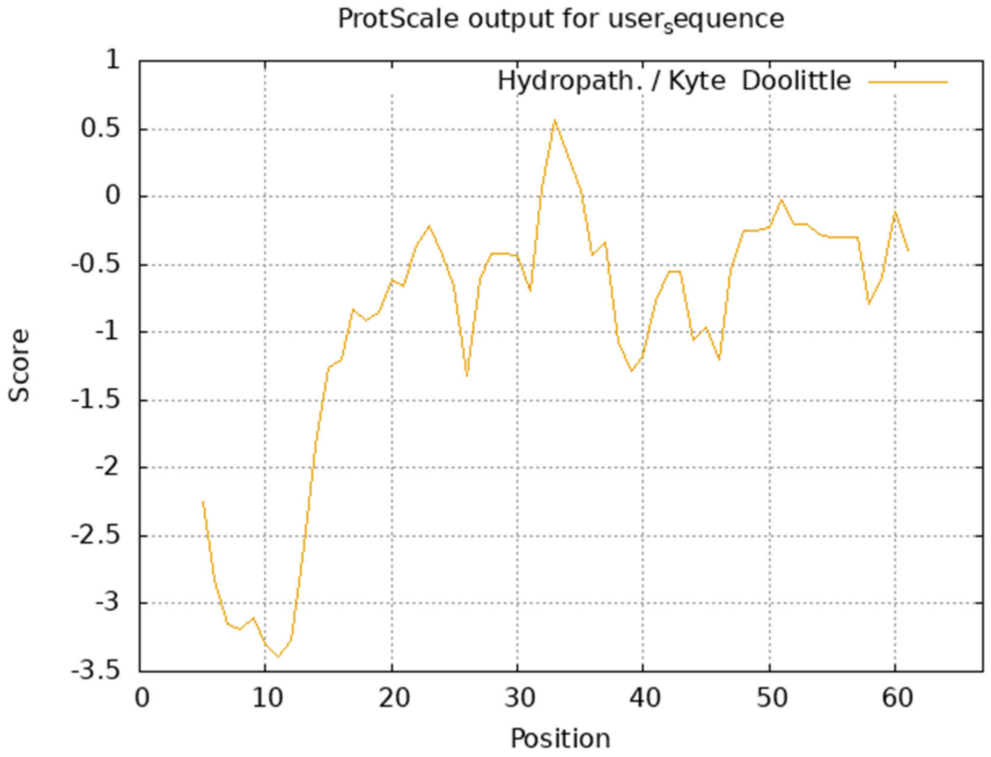

Figure S1. Kyte–Doolittle hydropathy profile of *Urechis unicinctus* lumbricin predicted using ProtScale.

Notes: X-axis, amino acid residue position; Y-axis, hydropathy score. Positive values indicate hydrophobic regions, and negative values indicate hydrophilic regions.
