# Supplementary material for: Molecular Cloning and Functional Analysis of Lumbricin in Urechis unicinctus
Source: Biology (Basel). 2026 Sep 9;15(18):1590. doi: 10.3390/biology15181590 (PMC13603904; doi:10.3390/biology15181590)
Supplement: Supplementary file 1 [file biology-15-01590-s001.zip › Figure S2.pdf]

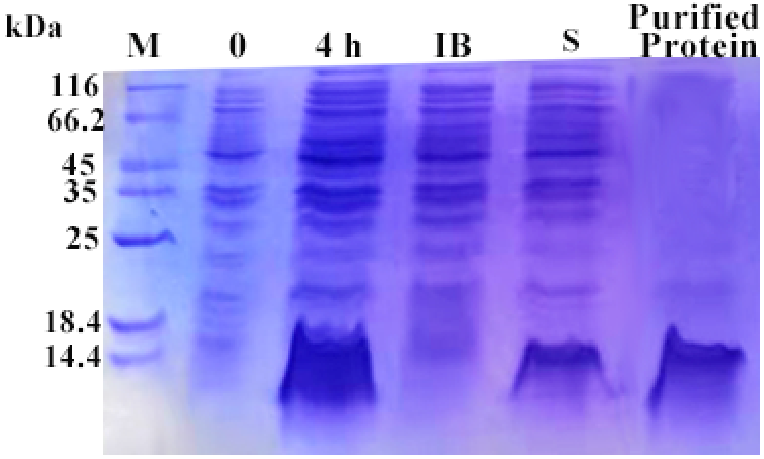

Figure S2. SDS-PAGE detection of recombinant lumbricin expression and purification. M: low-molecular-weight protein marker (116.0, 66.2, 45, 35, 25, 18.4, 14.4 kDa); 0: uninduced bacterial control; 4 h: 4 h IPTG-induced whole-cell lysate; IB: inclusion bodies; S: soluble supernatant; Purified Protein: Ni-NTA purified target protein.
