# Supplementary material for: Molecular Cloning and Functional Analysis of Lumbricin in Urechis unicinctus
Source: Biology (Basel). 2026 Sep 9;15(18):1590. doi: 10.3390/biology15181590 (PMC13603904; doi:10.3390/biology15181590)
Supplement: Supplementary file 1 [file biology-15-01590-s001.zip › Table S1.pdf]

Table S1. SignalP 6.0 prediction of N-terminal signal peptides for all lumbricin homologs

No.	Species	Sequence name	GenBank accession	Signal peptide prediction
1	*Eisenia andrei*	Lumbricin	ARR73564.1	Absent
2	*Eisenia andrei*	Lumbricin-related peptide	ARR73565.1	Absent
3	*Hirudo medicinalis*	Lumbricin	ABW97520.1	Absent
4	*Lumbricus rubellus*	Lumbricin-1	AAC64517.1	Absent
5	*Metaphire tschiliensis*	Lumbricin	AAO16887.1	Absent
6	*Metaphire guillelmi*	Lumbricin	P86929	**Present**
7	*Urechis unicinctus*	Lumbricin (this study)	—	Absent

Signal peptides were predicted using SignalP 6.0 with default parameters. Only 1 of 7 (14.3%) lumbricin homologs carries a canonical N-terminal signal peptide, confirming that signal-peptide deficiency is the predominant feature of the lumbricin family.
